# Cortical oscillations that underlie visual selective attention are abnormal in adolescents with cerebral palsy

**DOI:** 10.1038/s41598-021-83898-3

**Published:** 2021-02-25

**Authors:** Rashelle M. Hoffman, Christine M. Embury, Brandon J. Lew, Elizabeth Heinrichs-Graham, Tony W. Wilson, Max J. Kurz

**Affiliations:** 1 Institute for Human Neuroscience, Boys Town National Research Hospital, 14000 Boys Town Hospital Road, Boys Town, NE 68010 USA; 2 Department of Physical Therapy, Munroe-Meyer Institute, University of Nebraska Medical Center, Omaha, NE USA

**Keywords:** Cognitive neuroscience, Motor control

## Abstract

Adolescence is a critical period for the development and refinement of several higher-level cognitive functions, including visual selective attention. Clinically, it has been noted that adolescents with cerebral palsy (CP) may have deficits in selectively attending to objects within their visual field. This study aimed to evaluate the neural oscillatory activity in the ventral attention network while adolescents with CP performed a visual selective attention task. Adolescents with CP (N = 14; Age = 15.7 ± 4 years; MACS I–III; GMFCS I–IV) and neurotypical (NT) adolescents (N = 21; Age = 14.3 ± 2 years) performed the Eriksen flanker task while undergoing magnetoencephalographic (MEG) brain imaging. The participants reported the direction of a target arrow that was surrounded by congruent or incongruent flanking arrows. Compared with NT adolescents, adolescents with CP had slower responses and made more errors regarding the direction of the target arrow. The MEG results revealed that adolescents with CP had stronger alpha oscillations in the left insula when the flanking arrows were incongruent. Furthermore, participants that had more errors also tended to have stronger alpha oscillatory activity in this brain region. Altogether these results indicate that the aberrant activity seen in the left insula is associated with diminished visual selective attention function in adolescents with CP.

## Introduction

Cerebral palsy (CP) stems from a perinatal brain insult and is the most common pediatric motor disability, impacting 3.1 per 1000 live births in the United States^1,2^. In addition to the motor impairments, some individuals with CP have visual attention deficits compared to their age matched peers^3^. Adolescence is a key developmental period for the refinement of several cognitive functions, including visual perception^4^. Such maturational changes in visual perceptual processes are paralleled by developmental shifts in the frontal lobes, a region strongly associated with attention and executive processes^5^. The current view is that the impairments in visual perception often seen in adolescents with CP may result from acuity, retinopathy of prematurity, and/or strabismus^6,7^. Such visual deficits may disrupt the ability to selectively attend to a visual stimulus while performing goal-oriented motor behaviors. Although this is plausible, this conjecture has not been specifically tested.

The ability to focus on a target visual stimulus while disregarding distracting or unrelated stimuli is central to visual selective attention function^8,9^. The classic Eriksen flanker task has been widely utilized to probe visual selective attention^10^. This task requires participants to focus on a target visual stimulus and disregard the surrounding “flanker” stimuli. The task includes trials with flanking stimuli that are identical to the target stimuli (e.g., arrows pointing the same direction; congruent trials) and trials where the flanking stimuli are the opposite of the target stimuli (e.g., arrows pointing in the opposite direction; incongruent trials). Behaviorally, this task elicits a robust difference commonly termed the “flanker effect,” whereby participants are less accurate and/or respond more slowly on incongruent compared to congruent trials, which is interpreted as an inability to fully suppress the interference caused by the distracting, incongruent flanking arrows^11,12^. Magnetoencephalographic (MEG) brain imaging has indicated that there are changes in the strength of the theta (4–8 Hz) and alpha (8–12 Hz) cortical oscillations in the temporoparietal junction, the ventral frontal cortex, and the insula while performing this task^11–16^. These neural generators comprise the ventral attention network, which is thought to facilitate stimulus detection amidst distracting stimuli. Despite the growing recognition of the role of the ventral attention network, we have limited knowledge about the function of this network in individuals with CP.

This investigation used MEG brain imaging and advanced image reconstruction methods to evaluate cortical oscillations within the ventral attention network as adolescents with CP performed an arrow-based version of the flanker visual selective attention task. We hypothesized that (1) the visual attention-related cortical oscillations would be aberrant in adolescents with CP compared with neurotypical (NT) adolescents, and (2) that these atypical cortical oscillations would be tightly coupled with the speed and accuracy of their behavioral response on the direction of the target arrow.

## Methods

### Participants

NT adolescents and those with CP participated in this cross-sectional investigation. The adolescents with CP were referred from staff occupational and physical therapists at the Munroe-Meyer Institute who provide educational services. The adolescents with CP included in this study had primarily white matter injuries and no volume loss that would affect the integrity of the cortical surface. Adolescents were excluded from participating in this study if their cognitive function was too low to complete the Eriksen Flanker task, they had metal in their body that would preclude the use of MEG, or prior surgery and/or botulinum toxin injections in the past 6 months. Each adolescent and their parent/guardian provided written and informed assent/consent, respectively, to participate in the investigation. The protocol for this investigation was approved by University of Nebraska Medical Center’s Institutional Review Board and in compliance with the Code of Ethics of the World Medical Association. Effectively, all of the methods used in this investigation were performed in accordance with the relevant guidelines and regulations.

### Experimental design

Of note, the methodology employed in the current investigation is similar to what has been utilized in our prior experimental studies^11,12,16–27^.

**Figure 1:**
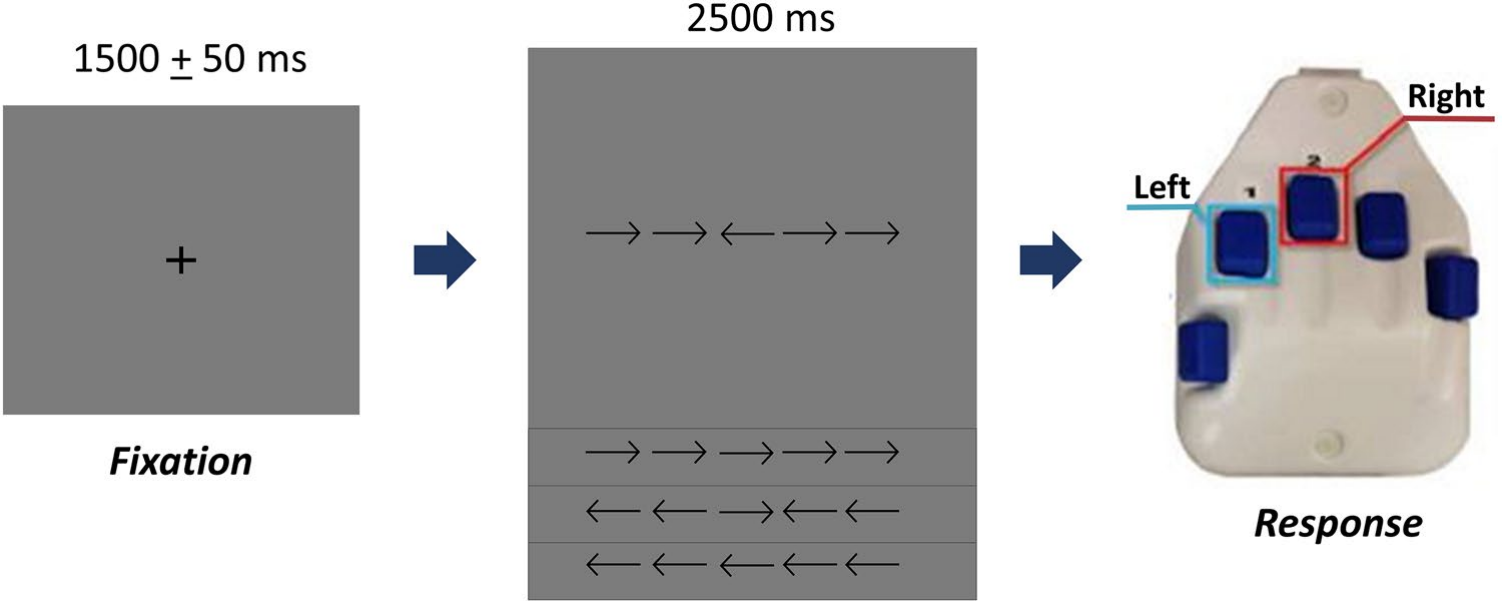
The Eriksen Flanker task paradigm. For each trial, the adolescents fixated on a crosshair for 1500 + 50 ms, then a display with a series of five arrows appeared for 2500 ms. The adolescents responded with their right hand regarding the direction of the center arrow (left: 2nd digit, right: 3rd digit). In the congruent condition, the flanking arrows pointed in the same direction as the middle arrow, whereas in the incongruent condition the flanking arrows pointed in the opposite direction.

Neuromagnetic responses were sampled continuously at 1 kHz with an acquisition bandwidth of 0.1–330 Hz using a MEGIN MEG system (Helsinki, Finland). The participants completed an arrow-based version of the Eriksen flanker task^10^. Each trial began with a fixation cross that was presented for an interval of 1500 ± 50 ms. A row of five arrows was then presented for 2500 ms and the adolescents were instructed to respond as to the direction of the center target arrow with their second (left arrow) or third (right arrow) digit of the right hand using a custom 5-button pad (Fig. 1). Visual presentation consisted of either a series of flanking arrows that had directions that were congruent (i.e., same direction) or incongruent (i.e., opposite direction) with the middle target arrow. The task stimuli was projected onto a screen that was approximately one meter from the participant. A total of 200 trials were presented, making the overall MEG recording time about 14 min. Trials were equally split and pseudo-randomized between congruent and incongruent conditions, with left and right pointing arrows being equally represented in each condition. Only correct responses were included for further analysis.

### MEG coregistration, MEG pre-processing and source imaging

Four coils were affixed to the participant’s head and were used for continuous head localization. Prior to the experiment, the location of these coils, three fiducial points, and the scalp surface were digitized to determine their three-dimensional position (Fastrak 3SF0002, Polhemus Navigator Sciences, Colchester, VT, USA). The coil locations during the MEG acquisition and the scalp surface points were used to coregister the MEG data with the native space neuroanatomical MRI data.

Using the MaxFilter software (MEGIN), each MEG dataset was individually corrected for head motion that may have occurred during task performance and was subjected to noise reduction using the signal space separation method with a temporal extension^28^. Artifact rejection was based on a fixed threshold method, supplemented with visual inspection. The continuous magnetic time series was divided into epochs of 2000 ms in duration, with 0 ms defined as the stimulus onset and the baseline being − 450 to − 50 ms. Artifact-free epochs for each sensor were transformed into the time–frequency domain using complex demodulation and averaged over the respective trials. These sensor-level data were then normalized per time–frequency bin using the mean power for the specific frequency during the baseline (− 450 to − 50 ms). The specific time–frequency windows used for imaging were determined by statistical analysis of the sensor-level spectrograms across the entire array of gradiometers. The specific time–frequency windows used for imaging were determined by statistical analysis of the sensor-level spectrograms across the entire array of gradiometers. This involved performing 1000 cluster based permutation paired-sample t-tests to define time–frequency bins containing potentially significant oscillatory deviations^29,30^. Note that the determination of the time–frequency windows to image was based on the spectrograms created from the entire group and both Flanker conditions. Additional information on our data processing pipeline is available in Weisman and Wilson^31^.

A beamforming algorithm was employed to calculate the source power across the entire brain volume^32^. The single images were derived from the cross spectral densities of all combinations of MEG sensors, and the solution of the forward problem for each location on a grid specified by input voxel space. Following convention, the source power in these images was normalized per subject using a separately averaged pre-stimulus noise period of equal duration and bandwidth^33–35^. Thus, the normalized power per voxel (pseudo-t) was computed over the entire brain volume per adolescent at 4.0 × 4.0 × 4.0 mm resolution. Each adolescent’s functional images, which were co-registered to their structural T1-weighted MRI prior to beamforming, were transformed into standardized space using the transform previously applied to the structural MRI volume and spatially resampled. MEG pre-processing and imaging used the BESA software (BESA v6.0; Grafelfing, Germany). A whole-brain repeated-measures ANOVA (congruent/incongruent x group) was subsequently completed for each time–frequency window of interest to determine locations of significant voxel clusters.

### Motor behavioral data

The output of the button pad was simultaneously collected at 1 kHz along with the MEG data. The time the participant took to decide the direction of the target arrow (i.e., reaction time) was calculated based on the time from when the arrow array was presented to when the button was pressed. Accuracy was defined as the percentage of correct responses divided by the total number of trials. Separate repeated-measures ANOVAs (congruent/incongruent x group) were used to evaluate differences in the respective behavioral outcome measures. Lastly, Spearman rho rank-order correlations were used to determine potential relationships between behavioral and neuronal outcome metrics.

## Results

Fourteen adolescents with CP (Age = 15.7 ± 4.0 years; MACS levels I–III) and twenty-one NT adolescents (Age = 14.3 ± 2.0 years) fit the inclusion criteria and participated in this investigation. Further information on the respective participants can be found in Table 1.

**Table 1:** Participant demographics.

	Cerebral palsy (N = 14)	Neurotypical (N = 21)
Age (years)	15.7 ± 4.0	14.3 ± 2.0
Gender (male)	7	13
**MACS**		N/A
I	4	
II	7	
III	3	
**Type of cerebral palsy**		N/A
Diplegic	11	
Hemiplegic	3	

Participant demographics are displayed for the group with CP and the NT group.

Abbreviations: manual ability classification system (MACS).

### Behavioral results

**Figure 2:**
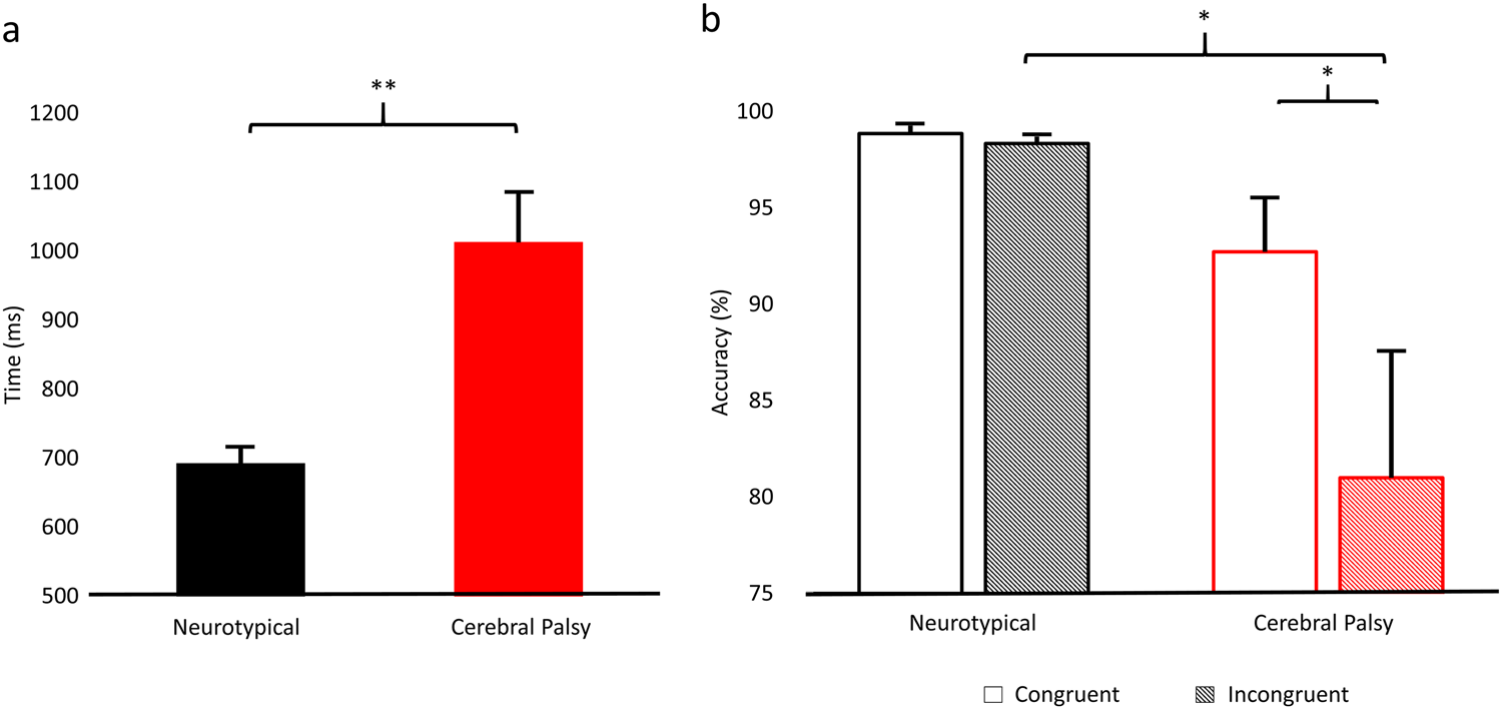
Reaction time and accuracy for neurotypical adolescents and adolescents with cerebral palsy. Repeated-measures analysis of variance of reaction time (**a**) and accuracy (**b**). Reaction time results revealed significant main effects of condition and group (*P* < 0.001), but no interaction effect (*P* = 0.4). The main effect of condition indicates slower reaction times for the incongruent condition compared to the congruent condition, while the main effect of group indicates that individuals with cerebral palsy responded slower than the neurotypical group. (**a**) Depicts the group effect (*P* < 0.001). Accuracy results revealed main effects of condition (*P* = 0.006), group (*P* = 0.004), and an interaction effect (*P* = 0.009). The main effect of condition reflected less accuracy for the incongruent compared to the congruent condition, while the main effect of group indicated that those with cerebral palsy were less accurate than the neurotypical adolescents. Post-hoc significance levels are displayed above the data, with error bars indicating the SEM. *P < 0.05, **P < 0.001.

There was a significant main effect of condition (congruent vs. incongruent) for the reaction time, which is consistent with the well-established “flanker effect” indicating that the adolescents took longer to respond during the incongruent compared to the congruent condition, likely due to the visually distracting flanking arrows (congruent = 785.3 ± 38.6 ms, incongruent = 849.7 ± 40.5 ms; *P* < 0.001). Additionally, there was a main effect of group, signifying that the adolescents with CP had slower reaction times relative to the NT adolescents across both conditions (CP = 1054.2 ± 78.4 ms, NT = 692.3 ± 23.9 ms; *P* < 0.001; Fig. 2a.). The group-by-condition interaction for reaction time was not significant (*P* = 0.4).

A Shapiro–Wilk test of the accuracy data indicated the sample data was not normally distributed (*P* < 0.001). Therefore, the accuracy data was log-transformed for statistical analysis. We found a main effect of condition (*P* = 0.006), indicating that adolescents were less accurate during the incongruent condition (congruent = 96.7 ± 1.1%; incongruent = 93.5 ± 2.2%; Fig. 2b). Additionally, there was a main effect of group (*P* = 0.004), indicating that the adolescents with CP were less accurate overall relative to the NT adolescents (CP = 87.5 ± 3.9%; NT = 99.9 ± 0.2%). Lastly, there was a significant group-by-condition interaction (*P* = 0.009). Post-hoc analysis showed that the adolescents with CP were significantly less accurate for the incongruent condition (incongruent = 81.0% ± 6.5%; congruent = 92.7% ± 2.8%; *P* = 0.037), while this effect was trending in the NT adolescents (incongruent = 98.3% ± 0.3%; congruent = 98.9% ± 0.4%; *P* = 0.099). When comparing within each condition, the adolescents with CP were significantly less accurate than the NT adolescents in the incongruent condition (CP = 81.0 ± 6.5%; NT = 98.3 ± 0.3%; *P* = 0.027) and marginally so for the congruent condition (CP = 92.7 ± 2.8%; NT = 98.9 ± 0.4%; *P* = 0.055).

### Sensor level results

**Figure 3:**
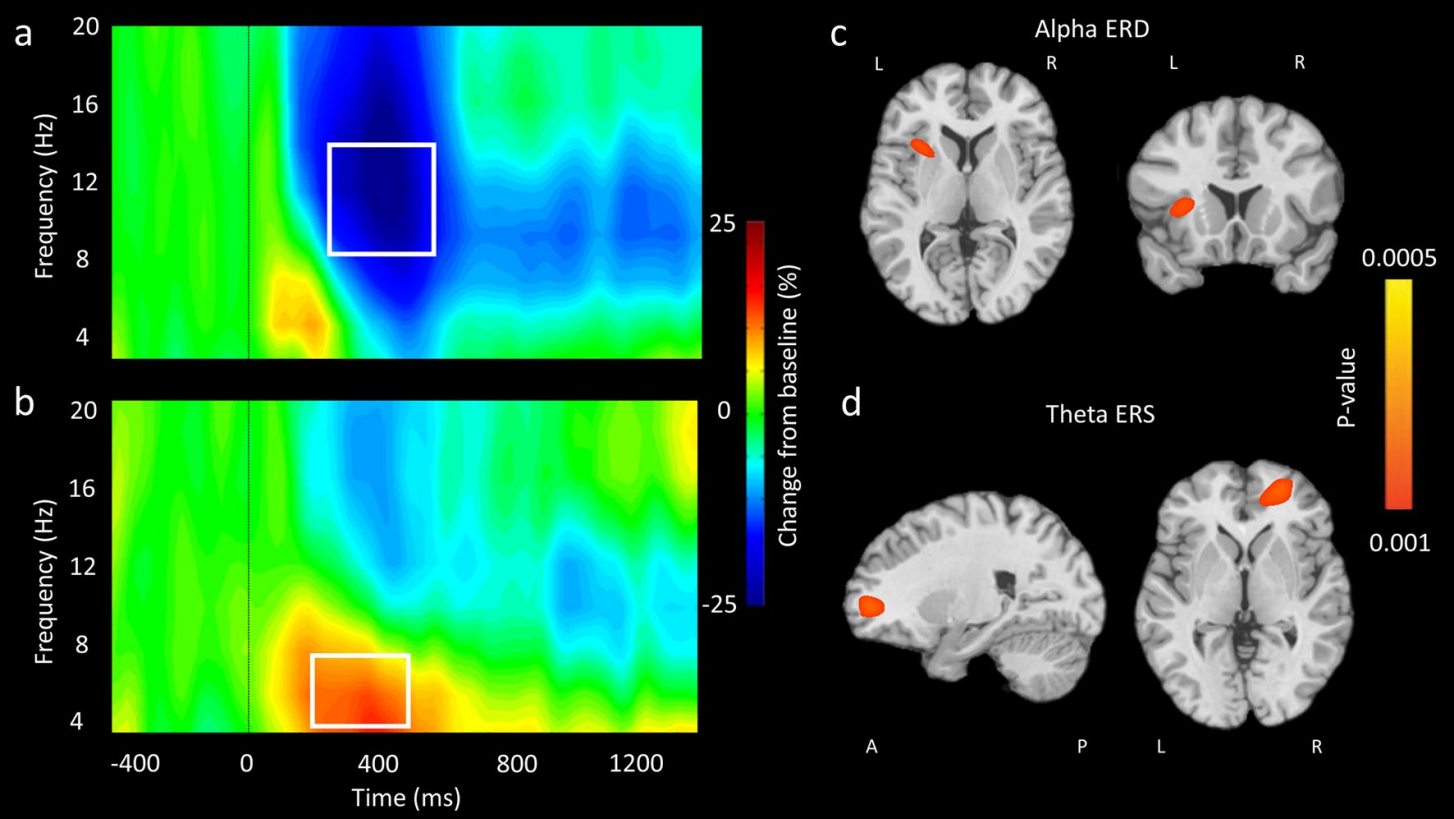
MEG sensor-level activity and whole brain repeated-measures ANOVA results for alpha ERD and theta ERS responses. MEG sensor-level grand-averaged spectrograms for the alpha ERD response in a sensor located over the parietal cortex (**a**), and the theta ERS response in a sensor located over the frontal cortex (**b**). In each, time (ms) is represented on the x-axis with 0 ms indicating stimulus onset (i.e., when arrows appeared). Relative spectral power is expressed as a percentage difference from baseline, with the color legend shown to the right of the spectrograms. As shown, there was a prominent alpha ERD (8–14 Hz) that began about 250 ms after stimulus onset and lasted until 650 ms. Additionally, there was a theta ERS (4–8 Hz) that began 200 ms after stimulus onset and was sustained until 600 ms. Whole-brain repeated-measures ANOVAs (condition x group) revealed an interaction for the alpha oscillations (8–14 Hz) in the left insula (**c**), while the same analysis for theta oscillations (4–8 Hz) showed an effect in the right middle frontal gyrus (**d**). All source images were created using NeuroElf V1.1(https://neuroelf.net/).

Time frequency analyses were conducted across all participants and gradiometer sensors and these were examined statistically to identify the time–frequency windows of interest for follow-up beamforming analysis. These analyses revealed significant alpha (8–14 Hz) event-related desynchronization (ERD) and theta (4–8 Hz) event-related synchronization (ERS) responses concentrated in a large number of sensors within the frontal cortices and stretching towards the parietal lobule (*P* < 0.0001, corrected). Desynchronizations indicate a decrease in power while synchronizations indicate an increase in power compared to the baseline window (− 450 to − 50 ms). For illustrative purposes, we show group-averaged time frequency spectrograms collapsed across groups and conditions in Fig. 3. The sensor-level statistical analyses indicated that there was a prominent alpha ERD about 250 ms after stimulus onset that extended until 650 ms (i.e., 250–650 ms; Fig. 3a). Additionally, there was a theta ERS that began about 200 ms after stimulus onset (0 ms) and was sustained until 600 ms (i.e., 200–600 ms; Fig. 3b).

### MEG imaging of cortical oscillations

Beamforming was used to image the significant alpha (8–14 Hz) response identified in the sensor level spectrograms during the 250–650 ms time window with a baseline of − 450 to − 50 ms. A whole-brain repeated-measures ANOVA (condition × group) was conducted with an α level of 0.001 and a cluster threshold of 200 voxels. This analysis revealed a significant condition effect in the right inferior frontal gyrus, indicating that there was less of a reduction in the strength of the alpha oscillations during the incongruent condition. Furthermore, there was an interaction in the left insula (Fig. 3c). Post-hoc analyses performed for the interaction showed that there was a stronger alpha ERD in the left insula for the adolescents with CP (*P* = 0.004) and NT adolescents (*P* = 0.02) during the incongruent compared to the congruent condition, and that the adolescents with CP had a stronger alpha ERD compared to NT adolescents in the incongruent (*P* = 0.02), but not the congruent condition (*P* = 0.7) in this cortical region.

Beamforming was also used to image the significant theta (4–8 Hz) response using the identified 200–600 ms time window and a baseline period of − 450 to − 50 ms. These data were also examined using whole-brain repeated-measures ANOVA (condition × group) approach with an α level of 0.001 and a cluster threshold of 200 voxels. We found a significant condition effect in the right dorsal lateral prefrontal cortices, left premotor cortices, and anterior cingulate. This indicated that there was a greater increase in the strength of the theta oscillations during the incongruent condition for the respective areas. There also was an interaction in the anterior right middle frontal gyrus (Fig. 3d). Post-hoc statistical analyses revealed that both the adolescents with CP (*P* = 0.04) and NT adolescents (*P* = 0.008) had significantly stronger theta ERS for the incongruent compared to the congruent condition in this region. No significant differences were found between groups for congruent or incongruent conditions (*P* > 0.05).

### Neuro-behavioral correlations

**Figure 4:**
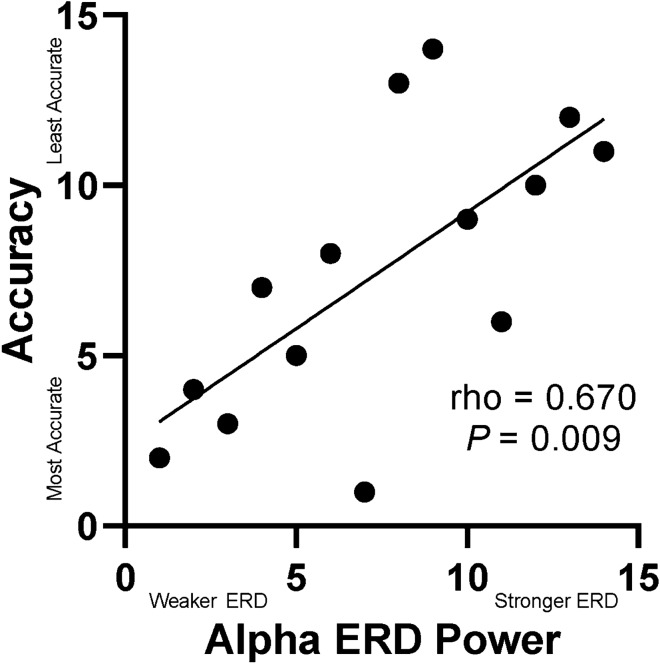
Rank-order correlation analysis between accuracy and alpha ERD Power. The alpha ERD peak voxel was identified in the left insula interaction effect map for the incongruent condition in the CP group only. Rank-order of the alpha ERD is shown on the x-axis, while the rank-order of accuracy is shown on the y-axis. As shown, there was a significant postive correlation between the strength of the change in the alpha ERD and the participant’s accuracy (rho = 0.670; *P* = 0.009). This correlation implies that adolescents with CP that had stronger alpha oscillations in the left insula also tended to make more errors in their perception of the target arrow’s direction. *ERD* event-related desynchronization.

There was a significant rank-order relationship between the strength of the alpha ERD in the left insula and accuracy (rho = 0.550, *P* = 0.001). This finding was for the incongruent condition only when collapsed across groups. Additionally, there was a significant rank-order relationship between the strength of the alpha ERD in the left insula and accuracy for the incongruent condition in the CP group only (rho = 0.670, *P* = 0.009; Fig. 4). Both correlations imply that the participants with a stronger alpha ERD tended to be less accurate in identifying the direction of the target arrow, which is consistent with our ANOVA results (see above). No other correlations between behavioral and neuronal outcomes measures were significant (*P* > 0.05).

## Discussion

Overall our experimental results for the NT adolescents and those with CP were well aligned with the prior MEG experimental outcomes using this task, which have shown there is an alpha ERD in the left insula and a theta ERS in the right middle frontal gyrus during task performance^11–16^. Furthermore, in line with previous studies, the neural oscillations for the incongruent condition were stronger than those detected in the congruent condition^16,17,36^. When we compared the strength of these neural oscillations between the respective groups, we identified that the adolescents with CP had a stronger alpha ERD than the NT adolescents in the left insula during the incongruent condition. Previous studies have suggested that a stronger alpha ERD reflects greater attentional demands created by the distracting incongruent arrows^11,12,16^. Based on this notion, we suspect that the stronger alpha ERD seen in the adolescents with CP might indicate that they utilize even greater cortical resources in attending to the visual stimulus amidst the distracting stimuli.

Compared with the NT adolescents, the adolescents with CP were slower to respond and made more errors in their perception of the target arrow’s direction. These behavioral results further support the notion that adolescents with CP have greater difficulty performing tasks that require selective visual attention. Our correlational results also indicated that participants that had greater behavioral errors also tended to have a stronger alpha ERD. Altogether these results further fuel the impression that the stronger alpha ERD observed in the adolescents with CP might represent greater difficulty in focusing on the target stimulus in the context of the distracting stimuli. We propose that the relationship observed between the alpha ERD and performance reflects that participants with CP who are poorer performers activate this region more strongly, but that ultimately this stronger activation is not enough to boost performance to normal levels. However, future studies are warranted as there could also be downstream effects that modulate performance, and/or aberrant activity in upstream brain regions that may be providing inferior information to this circuit necessitating that it work harder during task performance.

We recognize that there are limitations in our experimental approach. For one, the sample size for the adolescents with CP was relatively small and may not reflect the wide variety of neurologic impairments seen in individuals with CP. Second, there is growing appreciation that the timing of the perinatal insult may at least partially account for the nature of the structural brain differences (i.e., malformation, white matter injury) and possibly the variety of sensorimotor presentation seen in individuals with CP^37,38^. It is also possible that the type of perinatal brain insult may influence the activity of other nodes within the ventral attention network. Unfortunately, we cannot address this proposition since the participants in our study had white matter injuries. Furthermore, we are cautionary with this conjecture because the relationship between the type of brain insult and the resultant alterations in cortical activity is not well established. In summary, this study has provided new insight on the neural and behavioral aberrations that underlie selective attention deficits in adolescents with CP, but additional work in this area is critical.

## Data Availability

Data generated during the current study are available upon reasonable request and IRB approval.
